# Novel lncRNAs with diagnostic or prognostic value screened out from breast cancer via bioinformatics analyses

**DOI:** 10.7717/peerj.13641

**Published:** 2022-07-14

**Authors:** Hongxian Wang, Lirong Shu, Nan Niu, Chenyang Zhao, Shuqi Lu, Yanhua Li, Huanyu Wang, Yao Liu, Tianhui Zou, Jiawei Zou, Xiaoqin Wu, Yun Wang

**Affiliations:** 1 Department of Thyroid and Breast Surgery, Shenzhen Nanshan People’s Hospital and the 6th Affiliated Hospital of Shenzhen University, Shenzhen, Guangdong, P.R. China; 2 Longhua Innovation Institute for Biotechnology, College of Life Sciences and Oceanography, Shenzhen University, Shenzhen, Guangdong, P.R. China

**Keywords:** Breast cancer, Exosome, lncRNA, Biomarker, Diagnosis, Prognosis

## Abstract

**Background:**

Recent studies have shown that long non-coding RNAs (lncRNAs) may play key regulatory roles in many malignant tumors. This study investigated the use of novel lncRNA biomarkers in the diagnosis and prognosis of breast cancer.

**Materials and Methods:**

The database subsets of The Cancer Genome Atlas (TCGA) by RNA-seq for comparing analysis of tissue samples between breast cancer and normal control groups were downloaded. Additionally, anticoagulant peripheral blood samples were collected and used in this cohort study. The extracellular vesicles (EVs) from the plasma were extracted and sequenced, then analyzed to determine the expressive profiles of the lncRNAs, and the cancer-related differentially expressed lncRNAs were screened out. The expressive profiles and associated downstream-mRNAs were assessed using bioinformatics (such as weighted correlation network analysis (WGCNA), Gene Ontology (GO) and Kyoto Encyclopedia of Genes and Genome (KEGG) enrichments, Receiver-Operating Characteristic (ROC) curve and survival analysis, *etc.*) to investigate the diagnostic and prognostic values of these EV lncRNAs and their effectors.

**Results:**

In this study, 41 breast cancer-related lncRNAs were screen out from two datasets of tissue and fresh collected plasma samples of breast cancer via the transcriptomic and bioinformatics techniques. A total of 19 gene modules were identified with WGCNA analysis, of which five modules were significantly correlated with the clinical stage of breast cancer, including 28 lncRNA candidates. The ROC curves of these lncRNAs revealed that the area under the curve (AUC) of all candidates were great than 70%. However, eight lncRNAs had an AUC >70%, indicating that the combined one has a good diagnostic value. In addition, the results of survival analysis suggested that two lncRNAs with low expressive levels may indicate the poor prognosis of breast cancer. By tissue sample verification, C15orf54, AL157935.1, LINC01117, and SNHG3 were determined to have good diagnostic ability in breast cancer lesions, however, there was no significant difference in the plasma EVs of patients. Moreover, survival analysis data also showed that AL355974.2 may serve as an independent prognostic factor and as a protective factor.

**Conclusion:**

A total of five lncRNAs found in this study could be developed as biomarkers for breast cancer patients, including four diagnostic markers (C15orf54, AL157935.1, LINC01117, and SNHG3) and a potential prognostic marker (AL355974.2).

## Introduction

Breast cancer is the most common malignant tumor for women worldwide. It can result in great physical and mental harm to patients, a poor quality of life, and a heavy family and societal burden (Siegel, Miller & Jemal, 2018). The incidence of breast cancer has many complex high-risk factors and interactions (Plagens-Rotman et al., 2017). The breast cancer diagnosis may be missed or misdiagnosed when patients are in an early stage of the disease, since lesions at this point are heterogeneous, and patients may be without typical symptoms. Currently, *via* the popularization and promotion of routine screening, early diagnosis and effective treatments of breast cancer have been significantly improved, and the risk of patient death has been remarkably reduced (Ravert & Huffaker, 2010). Therefore, understanding the risk factors, drug targets, and the molecular pathogenesis and regulation mechanisms are of great interest in breast cancer research.

The rapid development of high-throughput technology in multi-omics has resulted in that the mining of biomarkers has important clinical value in early screening, differential diagnosis, and the precise treatment and prognosis of breast cancer. *BRCA1* and *BRCA2* have been shown to be biomarkers for predicting the familial hereditary of breast cancer (Lou et al., 2014). Estrogen receptor (ER) and progesterone receptor (PR) can be used as targets for the treatment of breast cancer to decide if the patients are suitable for endocrine therapy (Group EBCTC, 2005). Human epidermal growth factor receptor 2 (HER2) may also be used as a biomarker for the targeted therapy and prognosis of this disease (Ross et al., 2003). Many nucleic acids, proteins, and metabolites are candidates that may be involved in the carcinogenesis of breast cancer at different stages. These have been identified in preliminary research and have the potential for clinical application (Schwarzenbach & Pantel, 2015; Beretov et al., 2015; Speers et al., 2016).

LncRNA is one subtype of noncoding RNAs. LncRNA was thought to be made up of non-functional sequences that were produced during transcription. Recently, scientists have shown that lncRNAs may play the roles of “signal”, “guide”, “scaffold”, and “space occupying binding” in the process of transcription, post transcription, and epigenetic modification. LnRNAs are involved in the regulation of DNA methylation, histone modification, chromosome rearrangement, activation or silencing of target genes and other processing of molecular biology (Ponting & Belgard, 2010; Derrien, Guigó & Johnson, 2012; Wilusz, Sunwoo & Spector, 2009; Wang & Chang, 2011). Presently, many lncRNAs (such as H19, HOTAIR, MEG3, GAS5, and UCA1) have been shown to be closely related to the carcinogenesis of breast cancer (Su et al., 2014; Liu & Marie Pyle, 2015; Liu et al., 2013; Pickard & Williams, 2016; Huang et al., 2014). However, there are additional lncRNAs, that may play roles in the development of this disease.

High-throughput gene sequencing and microarray gene chip advancements have led to the establishment of The Cancer Genome Atlas (TCGA) and other public database platforms (Xu et al., 2015). The TCGA database can be used to search and mine biomarkers closely related to diseases. This tool has been used successfully to identify the regulatory mechanisms associated with some key targets linked to breast cancer lesions (Liu et al., 2018; Bhandari et al., 2018).

Extracellular vesicles (EVs) are discoid vesicles that exist throughout human organs and bodily fluids. EVs are approximately 40–1,000 nm in diameter with a lipid double-layer membrane. They contain nucleic acids, proteins, metabolites, and other molecules/substances. EVs are active organisms and are secreted from almost all cells under physiological and pathological conditions. They are involved in aspects such as the immune response, inflammatory response, cell differentiation, cell migration, tumor invasion, and metastasis (Boukouris & Mathivanan, 2015; He & Zeng, 2016; Gu, Hu & Li, 2017; Azmi, Bao & Sarkar, 2013; Whiteside, 2016). However, not much research has been conducted on the roles of plasma EV in breast cancer.

The weighted correlation network analysis (WGCNA) method helps to more comprehensively and systematically understand the occurrence and development of tumors. It has been widely used in various kinds of tumors including colon cancer, liver cancer, and glioblastoma multiforme (Zhai et al., 2017; Yin et al., 2018; Yang et al., 2018).

In this study, we attempted to find a number of key lncRNAs related to the carcinogenesis of breast cancer by comparing breast cancers to normal controls from the sequencing data of plasma EVs and tissue data from the TCGA database. We predicted their potential biological functions using WGCNA, and analyzed the function of those new biomarkers by Receiver-Operating Characteristic (ROC) curve and survival analyses to determine their diagnostic, therapeutic, and prognostic values in breast cancer.

## Materials & Methods

### Transcriptome sequencing data with clinical information collected from databases of breast cancer tissues

Using the TCGAbiolinks (Colaprico et al., 2016) R package, the breast cancer subdata of the transcriptome sequence from TCGA (the HTSeq-Counts type) were downloaded, resulting in a total of 1,222 breast tissue samples. The data from 794 cases of diagnosed invasive ductal carcinoma in breast tissues and 92 cases with normal tissue were also collected and downloaded with their clinical information.

### Screening of differentially expressed lncRNAs and mRNAs in breast cancer

The Deseq2 of R package was used to screen out the differentially expressed genes (DEGs) between the breast cancer and paracancerous tissue groups. |Log2 fold change| ≥ 1 and adjust *p* value <0.05 were applied as the selected threshold, and then the annotations were performed based on those candidates. The annotation films of Gencodev27.gtf in the GENCODE database (https://www.gencodegenes.org) served to distinguish the lncRNAs from RNA molecules.

### lncRNA sequencing data with correlation analyses between extracellular vesicles in plasma and tissues of breast cancer patients

The plasma samples of breast cancer and benign breast tumor groups (20 cases/group) for EV extracts were collected and treated from the 6th Affiliated Hospital of Shenzhen University, according to documents approved by the Medical Ethics Committee of the Union Shenzhen Hospital, Huazhong University of Science and Technology (0720001, ky-2020-039-02), and clinical trial registration (ChiCTR19002505). A written informed consent was obtained from every participant for this study.

Before RNA-seq, 10 plasma samples were pooled into one with an equal volume from same group, and the total RNAs of plasma were extracted using the exoRNeasy Serum/Plasma Maxi Kit (QIAGEN, USA). The large RNA molecules were sequenced to detect and analyze the expression levels of the lncRNAs.

The data were also compared with the expression of plasma EVs in other cancers from the exoRbase in order to find the disease-related lncRNAs in breast cancer. The lncRNA candidates of plasma EV were compared with lncRNAs of tissues, in order to screen out cancer-related lncRNAs, which were both differentially expressed in plasma EVs and breast tissues.

### Construction of weighted gene co-expression network

A weighted gene co-expression network analysis (WGCNA) was conducted (Langfelder & Horvath, 2008) to investigate the function of the lncRNAs by extracting their expression data from the TCGA and obtaining the clinical information from the samples. The data were processed and the function “goodSamplesGenes” was used to test the gene expression matrix to confirm whether there was a missing value. The “hclust” function was used to cluster the samples to remove the outlier samples. Using the Pearson correlation coefficient, the linear correlation degree between the two genes was analysed and the gene expression matrix was transformed into the gene relationship matrix. Then the inter-gene relationship matrix was transformed into a weighted scale-free network; the index *β* (soft threshold) was taken for the previously obtained Pearson correlation coefficient between genes, and the *β* value was adjusted to make the network meet the scale-free characteristics, resulting in the adjacency matrix of genes. The adjacency matrix was upgraded to a more rigorous and reliable topological overlap matrix (TOM). Using the difference degree of TOM as the clustering distance for cluster analysis, the last branch of the cluster tree represented genes, and the genes with a high overlap were clustered together to form large branches. Using the dynamic tree pruning (DynamicTreeCut), all genes were divided into different modules, and the number of genes contained in the module were not less than 30, and the modules were distinguished by colour (Langfelder, Zhang & Horvath, 2008). The genes in the module were highly related, and there were a small number of genes, which reflected the expression characteristics of the whole module. This was called the feature vector gene (Module eigengene, ME) of the module.

### Identification of the key lncRNA with clinical information

After the module identification was completed, it was combined with clinical information for analysis. The operation of WGCNA was based on continuous variables and classification variables, such as sample type (tumour or normal). The TNM was set to continuous variables. The sample type was converted to 0 or 1 min and the TNM staging was converted to the corresponding number (0, 1, 2, 3, 4, 5). If there was no clear clinical information in the sample, the samples were treated as a null value. Other clinical data such as age and survival time were not modified. The correlation between the modules and clinical features was achieved by calculating the Pearson correlation coefficient between ME and clinical features. Additional studies were performed to analyse the gene composition in the selected modules, identify the lncRNAs and the top five mRNAs related to lncRNAs, use Cytoscape to visualize the results, and apply g:profiler (Raudvere et al., 2019) for enrichment analysis to predict the potential biological functions of lncRNA in breast cancer.

### Assessment of biomarker function of core lncRNAs

The ROC curve was used to evaluate the diagnostic ability of the key lncRNAs to distinguish between breast cancer samples (*n* = 794) and normal samples (*n* = 92); the standard of screening was AUC area >70%. Univariate cox regression analysis was used in conjunction with the clinical information to study the relationship between the initial age at diagnosis, tumour stage, expression of key lncRNA, and total survival time. The initial age at diagnosis was categorized into a young and old group according to the median age. According to the tumour stage, stage I and II were classified as early stage, and stage III and greater were classified as late stage. The selected factors were then analysed by multivariate cox regression analysis to screen the factors with independent prognostic function.

### Partial verification of key lncRNA

The expression data of EV RNA (118 cases of normal and 140 cases of tumour) and tissue RNA (12 cases of normal and 74 cases of tumour) of breast cancer were downloaded from exoRBase and Gene Expression Omnibus database (GEO). Log _2_ (TPM+1) was used as the expression of key lncRNA, and the expression of key lncRNA was analysed using the Student’s *t*-test. ROC curves were then used to analyse the diagnostic roles of key lncRNAs in EVs and tissues, respectively.

### Statistical analysis

All data statistics were carried out by R software (version 3.5). The screening of differentially expressed genes (DEGs) was completed by R packet Deseq2. Genes with an |Log2 fold change | ≥1 and adjusted *p*-value <0.05 were considered to be differentially expressed. The resulting adjusted *p*-value was obtained using the Benjamini and Hochberg’s approach. The diagnostic ability and prognostic ability of lncRNA were performed by ROC curve (R packet pROC) and survival analysis (R packet survival and survminer), respectively. Univariate cox regression analysis and multivariate cox regression analysis were used to find lncRNA with independent prognosis; HR ≠ 1 and *p* < 0.05 were used as screening factors. In the verification analysis, the expression of lncRNA was treated with log2 (TPM+1), and the comparison of expression levels between normal tissues and tumors was analyzed using the Student’s *t*-test.

## Results

### Differential expression and gene annotation of lncRNAs in breast cancer tissues

**Figure 1:**
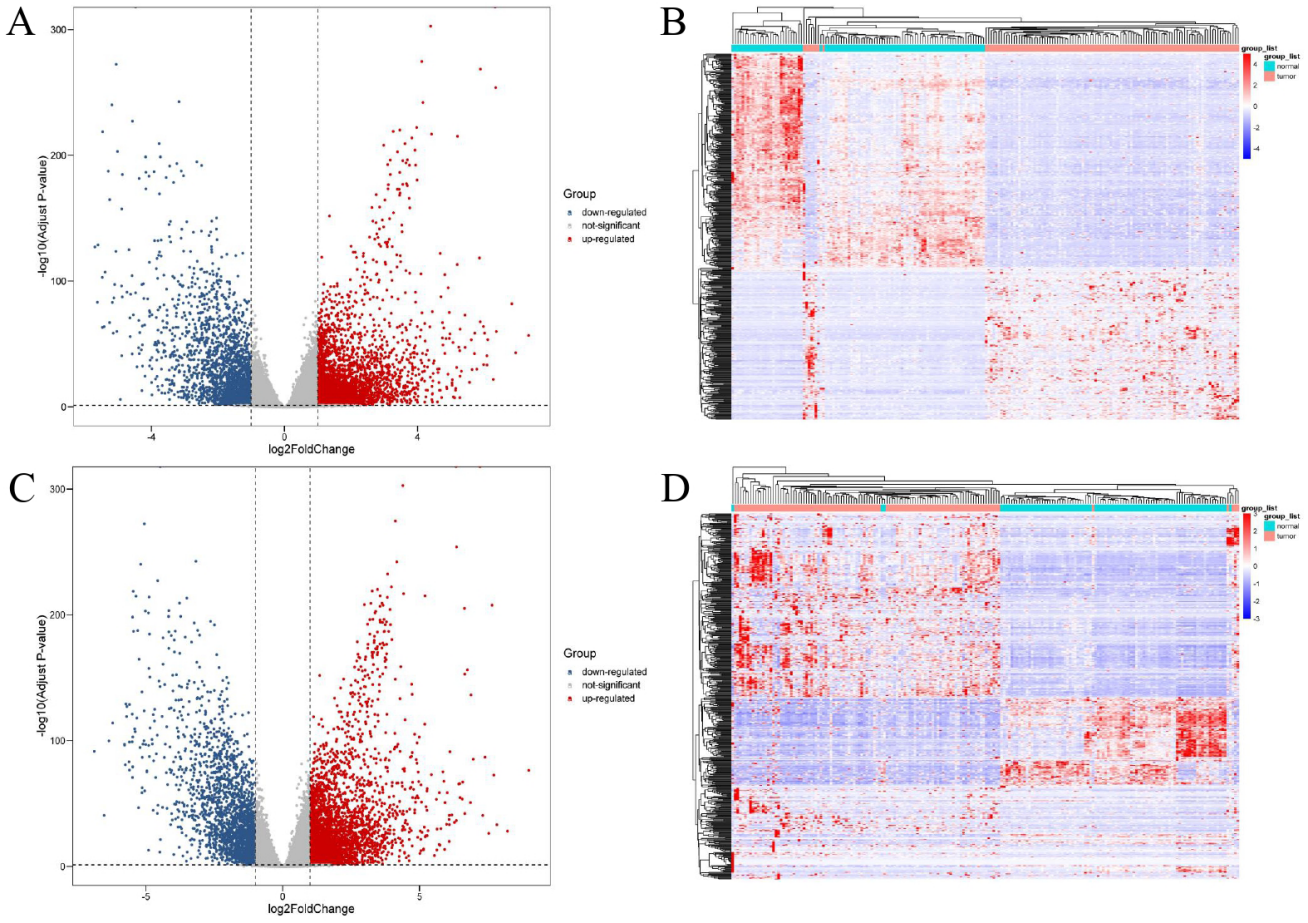
Expression profiles of lncRNAs and mRNAs in breast cancer and adjacent tissues. The red dots indicated that were up-regulation and blue dots pointed down-regulation. (A) Volcano plot of differentially expressed lncRNAs; (B) heat maps of differentially expressed lncRNAs; (C) volcano plot of differentially expressed mRNAs; (D) heat maps of differentially expressed mRNAs.

A total of 11,321 differentially expressed genes (|log2Fold Change| ≥ 1, adjusted *p*-value <0.05) were obtained from 56,512 genes of 886 tissue samples using DESeq2. These were combined with the annotation information in GENCODEv27.gtf and 4,879 differentially expressed lncRNAs and 5,304 differentially expressed mRNAs were obtained. Further study showed that 2,883 of these differentially expressed lncRNAs were up-regulated and 1,996 were down regulated. Of the differentially expressed mRNAs, 3,226 were up-regulated and 2,078 were down-regulated. The different expression patterns of lncRNAs and mRNAs are shown in Fig. 1.

### Comparative analysis of plasma lncRNAs in plasma EV of breast cancer patients

**Figure 2:**
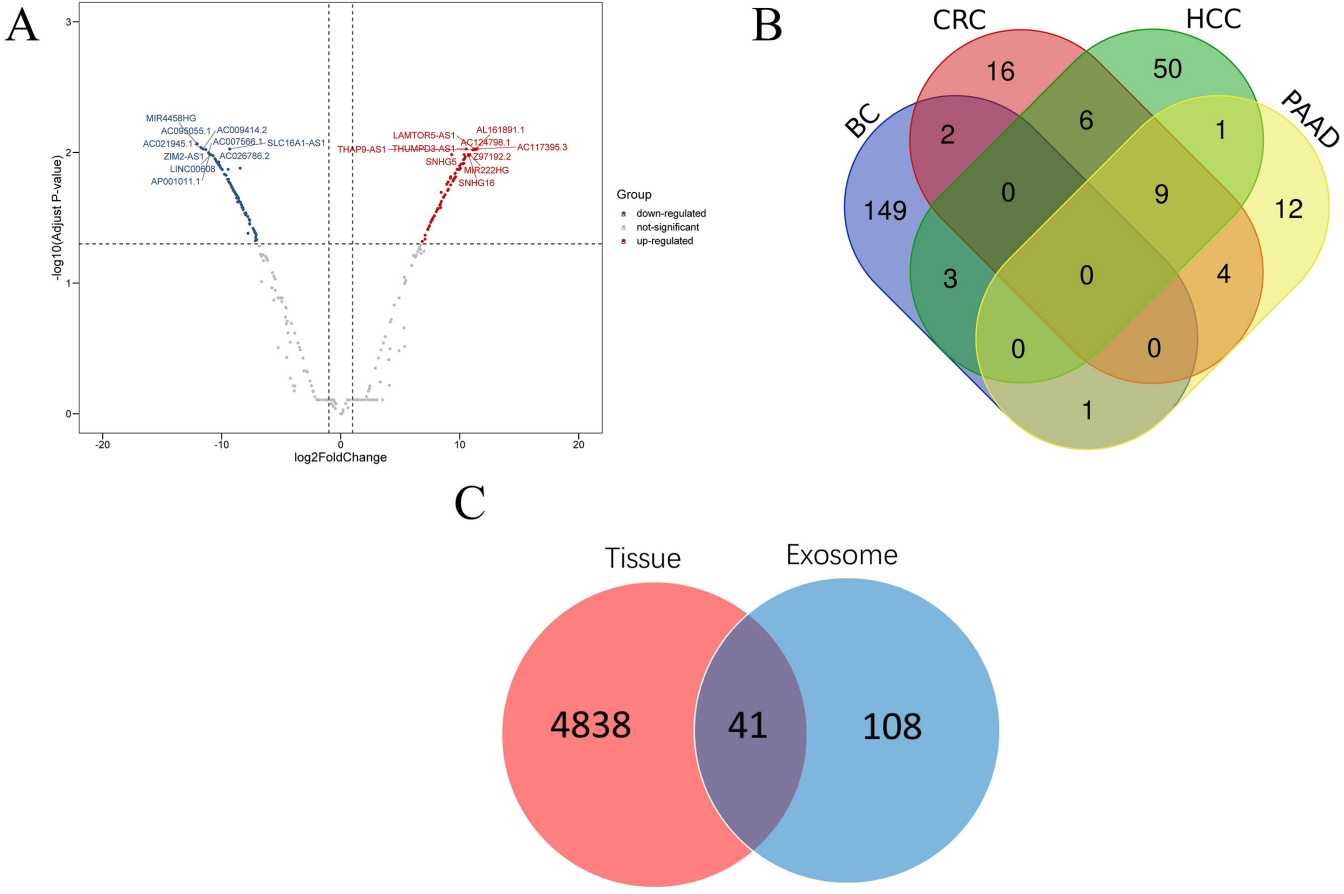
Disease related lncRNAs expression profile in plasma exosomes. (A) Volcano plot of differential expression of lncRNAs; (B) differential expression of lncRNAs of four kinds of cancer; (C) Venn diagram of differential expression of lncRNAs in breast cancer tissues and plasma EVs. Abbr: CRC, colon cancer; HCC, liver cancer; PAAD, pancreatic cancer; BC, breast cancer.

According to the threshold value of | log_2_Fold Change | ≥1 and adjust *p* value <0.05, a total of 2,714 DEGs were screened using DESeq2 software. Then 155 lncRNAs and 2,450 mRNAs were selected by annotating the differentially expressed genes. Among the differentially expressed lncRNAs, 72 were up-regulated and 83 were down-regulated. In addition, among the differentially expressed mRNAs, 1,619 were up-regulated and 831 were down regulated (Fig. 2). The top 20 differentially expressed lncRNAs are shown in Table 1. A total of 12 samples of blood extracellular vesicles (GSE100063) from patients with colon cancer (CRC), 21 samples of blood extracellular vesicles (GSE100207) from patients with hepatocellular carcinoma (HCC), and 12 samples of blood extracellular vesicles (GSE100207) from patients with pancreatic cancer (PAAD) were downloaded from the exoRBase database. Furthermore, the expression profiles of these samples were analysed using Limma package software, and lncRNAs were re-annotated by GENCODEv27.gtf. Thirty-seven differentially expressed lncRNAs were found in CRC, of which 33 were up-regulated and four were down-regulated; sixty-nine were found in HCC, of which 21 were up-regulated and 48 were down-regulated; a total of 28 were found in PAAD, of which 25 were up-regulated and three were down-regulated. A comparison of lncRNAs related to these diseases with those of the breast cancer lncRNAs showed that 149 lncRNAs were highly correlated with breast cancer. The data were further compared with lncRNAs in tissue expression profiles and 41 lncRNAs were found to be differentially expressed in both breast cancer tissues and plasma EVs.

**Table 1:** Differentially expressed lncRNAs at top 20 in plasma EV of breast cancer.

ID	Symbol	Biotype	log2FoldChange	Padjust
ENSG00000242791	AC117395.3	lincRNA	11.4652	0.0094
ENSG00000206573	THUMPD3-AS1	antisense_RNA	10.5516	0.0094
ENSG00000260196	AC124798.1	antisense_RNA	11.4021	0.0094
ENSG00000224699	LAMTOR5-AS1	processed_transcript	11.2664	0.0094
ENSG00000276672	AL161891.1	sense_intronic	11.2902	0.0095
ENSG00000213279	Z97192.2	lincRNA	11.3280	0.0095
ENSG00000251022	THAP9-AS1	antisense_RNA	11.1038	0.0096
ENSG00000203875	SNHG5	processed_transcript	9.3252	0.0104
ENSG00000163597	SNHG16	processed_transcript	10.7346	0.0104
ENSG00000270069	MIR222HG	lincRNA	10.8513	0.0104
ENSG00000247516	MIR4458HG	lincRNA	−12.0827	0.0086
ENSG00000270681	AC095055.1	antisense_RNA	−12.0568	0.0086
ENSG00000279347	AC021945.1	TEC	−11.7505	0.0092
ENSG00000226419	SLC16A1-AS1	antisense_RNA	−9.3304	0.0094
ENSG00000260025	AC009414.2	lincRNA	−11.5678	0.0094
ENSG00000269793	ZIM2-AS1	antisense_RNA	−11.3366	0.0095
ENSG00000244055	AC007566.1	antisense_RNA	−11.0982	0.0100
ENSG00000266049	AP001011.1	antisense_RNA	−11.0034	0.0104
ENSG00000236445	LINC00608	antisense_RNA	−10.9120	0.0104
ENSG00000271963	AC026786.2	antisense_RNA	−10.7291	0.0106

### Construction of weighted correlation network

**Figure 3:**
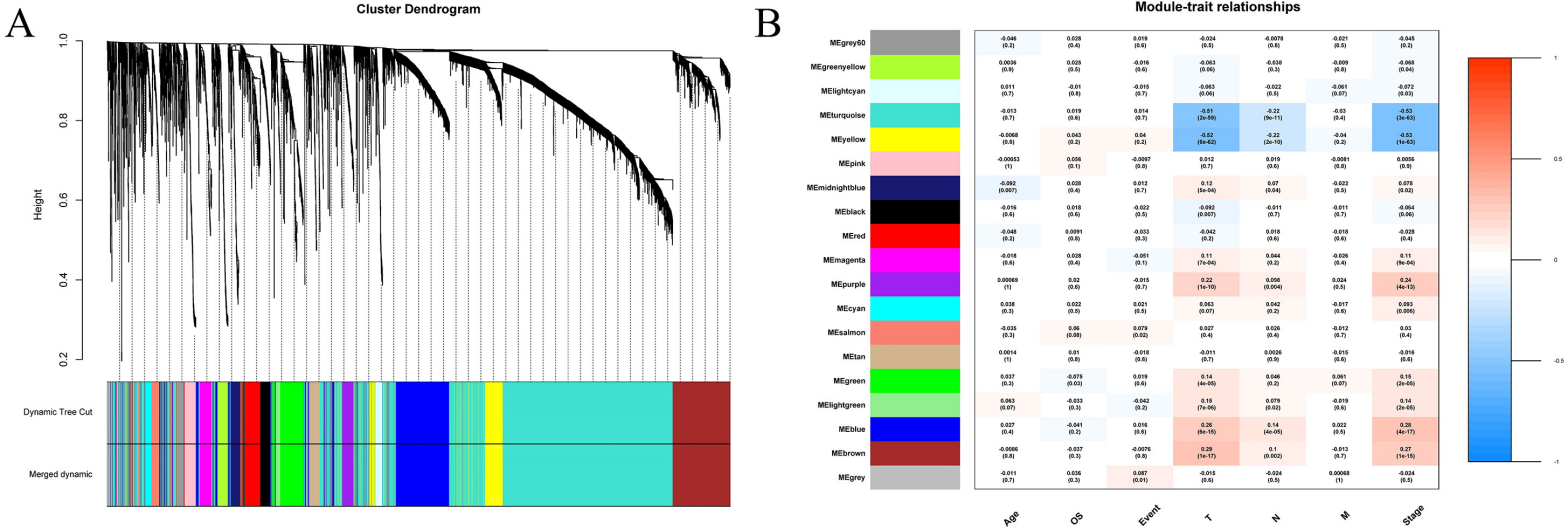
Construction of weighted correlation network. (A) Gene module cluster diagram was identified by dynamic pruning. The upper layer was the sample cluster tree, and the lower layer was the co-expression module of gene, and a total of 19 modules were obtained; (B) correlation heat maps between modules and different clinical characteristics. The abscissa is the clinical feature, the left ordinate is the module name, the right ordinate represents the threshold range of Pearson correlation coefficient, the correlation coefficients and *p* values of modules and traits are shown in the figures.

We extracted information on the expression of 41 lncRNAs and clinical information from the TCGA database for 866 tissue samples. This was integrated with the data previously obtained for 5,304 mRNA from the TCGA tissue samples to construct a weighted correlation network. The screening range threshold was defined as 0 to 30 and the correlation degree of log (k) and log (p (k)) under each threshold, as well as the corresponding average connectivity and average correlation degree were calculated. The soft threshold *β* = 3 met the requirements of scale-free network (Fig. S1). Therefore, the soft threshold = 3 was selected to calculate the adjacency matrix. The adjacency matrix was promoted to the TOM matrix, and the degree of TOM dissimilarity was used as the clustering distance for clustering analysis. A total of 5,345 genes were divided into different modules. As the number of genes contained in the module were not less than 30, and these modules were distinguished by the colour, a total of 19 gene modules were obtained. The Pearson correlation coefficient analysis module was used to analyse the correlation degree of each clinical feature and disease. Five modules (blue–green module, yellow module, purple module, blue module, and brown module) were found to have a relatively high correlation with the T stage and the TNM comprehensive stage of breast cancer (Fig. 3).

### Composition and function analyses of important modules

**Figure 4:**
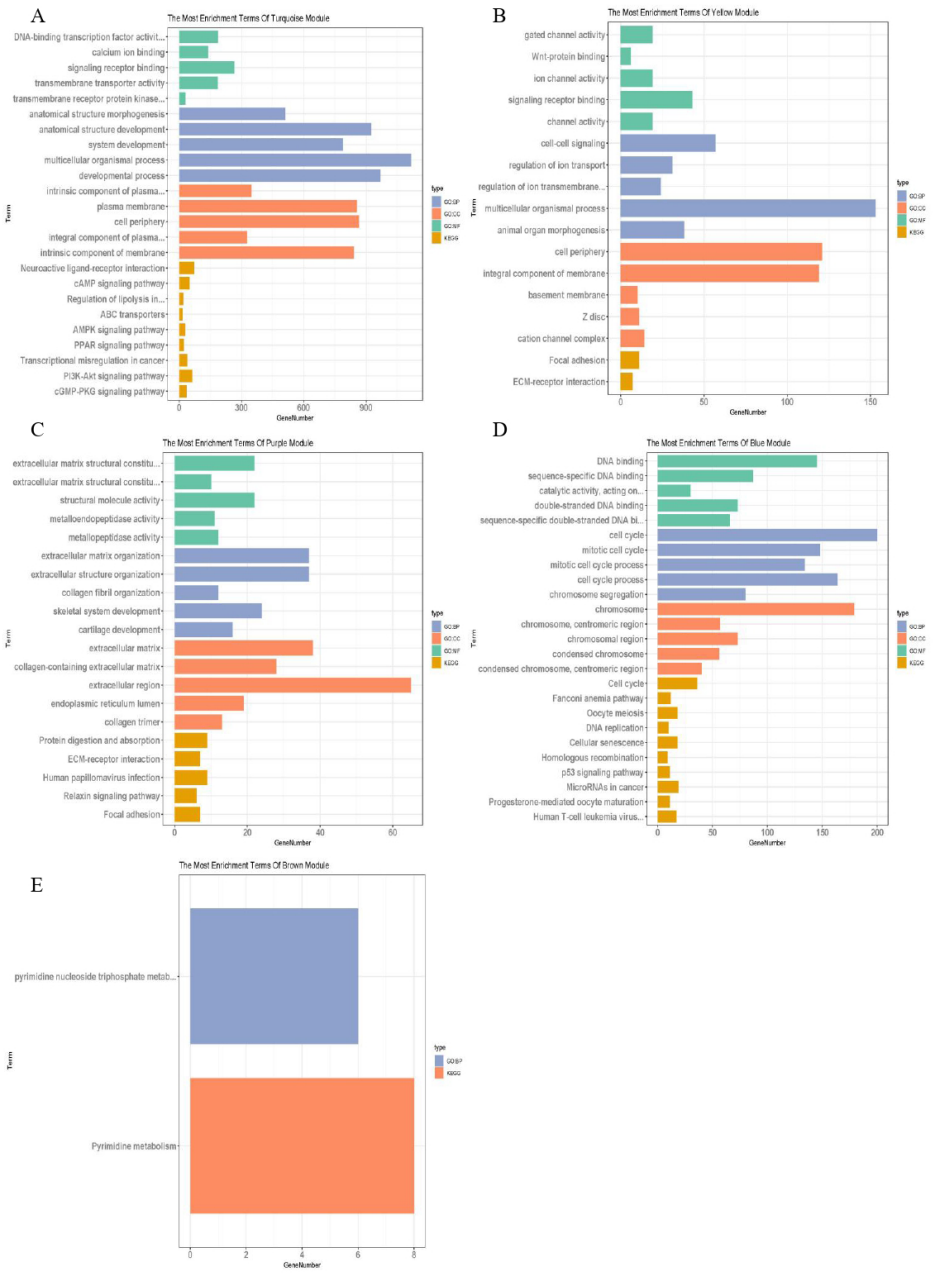
Function enrichment analysis of important modules. (A) Enrichment analysis of blue–green module; (B) enrichment analysis of yellow module; (C) enrichment analysis of purple module; (D) enrichment analysis of blue module; (E) enrichment analysis of brown module. The vertical axis represents the items of enrichment analysis, the horizontal axis represents the number of genes, and different colors represent BP, CC, MF, KEGG and other classifications.

The blue–green, yellow, purple, blue and brown modules related to TNM staging closely were analysed, and the lncRNAs and mRNAs with similar expression in the same module were identified, with a total of 28 of lncRNAs and 3,901 of mRNAs (Table 2). g: profiler was used to analyse the functional enrichment of important modules to explore their important role in breast cancer (Fig. 4). Among them, the genes of blue–green module were involved in the interaction of neuroactive ligand receptors and played roles in the cAMP signalling pathway, transcriptional imbalance in cancer, peroxisome proliferator activated receptors (PPARs) pathway, and PI3K Akt signalling pathway. The genes of yellow module might be involved in the formation of adhesive plaque and the interaction of extracellular matrix (ECM) receptors; the genes of purple module gene might be related DNA replication, cell cycle regulation, homologous recombination and the p53 signalling pathway related to the occurrence and development of breast cancer; while the genes of brown module might be involved in pyrimidine nucleotide metabolism and other processes. In the blue–green module, 19 lncRNAs and 2,273 mRNAs were obtained; in the yellow module, two lncRNAs and 289 mRNAs were obtained; in the purple module, 108 mRNAs were obtained; in the blue module, four lncRNAs and 658 mRNAs were obtained; in the brown module, three lncRNAs and 573 mRNAs were obtained.

**Table 2:** A list of important gene modules related to breast cancer with gene numbers.

Modules	Blue–Green	Yellow	Purple	Blue	Brown
LncRNA	19	2	0	4	3
mRNA	2273	289	108	658	573
Total	2292	291	108	662	576

**Figure 5:**
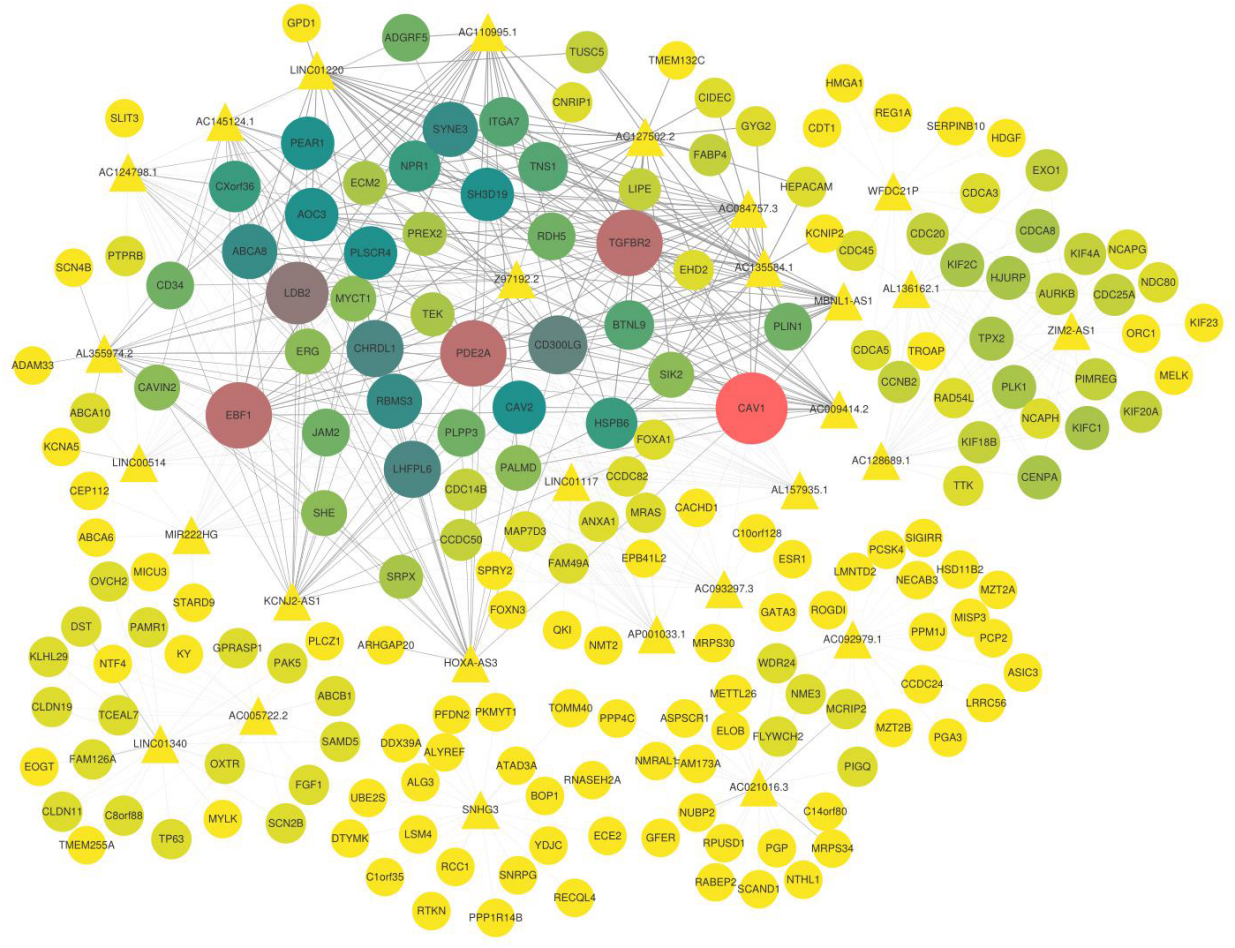
Co-expression network of lncRNAs and mRNAs. The triangle represents lncRNAs, the circle represents mRNA, the thickness of the line represents the strength of the correlation between lncRNA and mRNA, the size of the shape and the depth of the color represents the importance of mRNAs in the network. In addition, the shape is larger, the color is deeper, and the dot is more important in this network.

Furthermore, the candidate lncRNAs in the modules were further analysed and sorted by TOM value to find the mRNAs with a relatively high TOM value, which were the potential targeted regulatory genes of the lncRNAs. The top five were selected (Table 3), and the co-expression network was constructed according to the regulatory relationship among of them (Fig. 5). In the co-expression network, *TGFBR2*, *CAV1*, *PDE2A, LDB2, EBF1*, and other key genes were regulated by multiple lncRNAs.

**Table 3:** lncRNAs and matched mRNAs in the modules.

Module	lncRNA	mRNA				
blue–green	AC124798.1	*TGFBR2*	*LDB2*	*LHFPL6*	*PEAR1*	*RBMS3*
	AL355974.2	*LHFPL6*	*LDB2*	*TGFBR2*	*RBMS3*	*JAM2*
	AC084757.3	*EBF1*	*CAV1*	*AOC3*	*ABCA8*	*HSPB6*
	AC009414.2	*CAV1*	*PDE2A*	*CD300LG*	*EBF1*	*NPR1*
	AC093297.3	*MRPS30*	*FOXA1*	*TGFBR2*	*FAM49A*	*CAV2*
	MIR222HG	*STARD9*	*RBMS3*	*TGFBR2*	*LDB2*	*PLSCR4*
	AC110995.1	*CAV1*	*TGFBR2*	*CHRDL1*	*EBF1*	*ABCA8*
	AC145124.1	*LDB2*	*LHFPL6*	*TGFBR2*	*PEAR1*	*RBMS3*
	AP001033.1	*TGFBR2*	*LDB2*	*CAV1*	*EBF1*	*PDE2A*
	KCNJ2-AS1	*TGFBR2*	*LDB2*	*LHFPL6*	*RBMS3*	*PDE2A*
	AC135584.1	*AOC3*	*EBF1*	*PLIN1*	*HSPB6*	*CD300LG*
	HOXA-AS3	*LDB2*	*TGFBR2*	*LHFPL6*	*RBMS3*	*PDE2A*
	Z97192.2	*TGFBR2*	*LDB2*	*CAV1*	*EBF1*	*PDE2A*
	AL157935.1	*TGFBR2*	*CAV1*	*EBF1*	*LDB2*	*CAV2*
	LINC01117	*CCDC82*	*FAM49A*	*MRAS*	*CDC14B*	*CAV2*
	LINC01220	*AOC3*	*CAV1*	*EBF1*	*CD300LG*	*HSPB6*
	LINC00514	*KCNA5*	*TGFBR2*	*LDB2*	*RBMS3*	*PLSCR4*
	MBNL1-AS1	*CAV1*	*EBF1*	*AOC3*	*CHRDL1*	*HSPB6*
	AC127502.2	*EBF1*	*AOC3*	*PLIN1*	*HSPB6*	*CD300LG*
yellow	LINC01340	*DST*	*FAM126A*	*KLHL29*	*SCN2B*	*TCEAL7*
	AC005722.2	*DST*	*KLHL29*	*TCEAL7*	*SCN2B*	*GPRASP1*
blue	AL136162.1	*KIFC1*	*AURKB*	*HJURP*	*PLK1*	*TPX2*
	WFDC21P	*SERPINB10*	*CDC20*	*AURKB*	*KIFC1*	*PLK1*
	ZIM2-AS1	*CDCA8*	*HJURP*	*EXO1*	*TPX2*	*KIFC1*
	AC128689.1	*KIFC1*	*HJURP*	*TPX2*	*CDCA8*	*PLK1*
brown	AC021016.3	*NUBP2*	*MRPS34*	*MCRIP2*	*NME3*	*FAM173A*
	SNHG3	*UBE2S*	*ALYREF*	*LSM4*	*RNASEH2A*	*RTKN*
	AC092979.1	*PGA3*	*CCDC24*	*LMNTD2*	*PPM1J*	*PCSK4*

### Capability analysis of key lncRNAs as biomarkers

**Figure 6:**
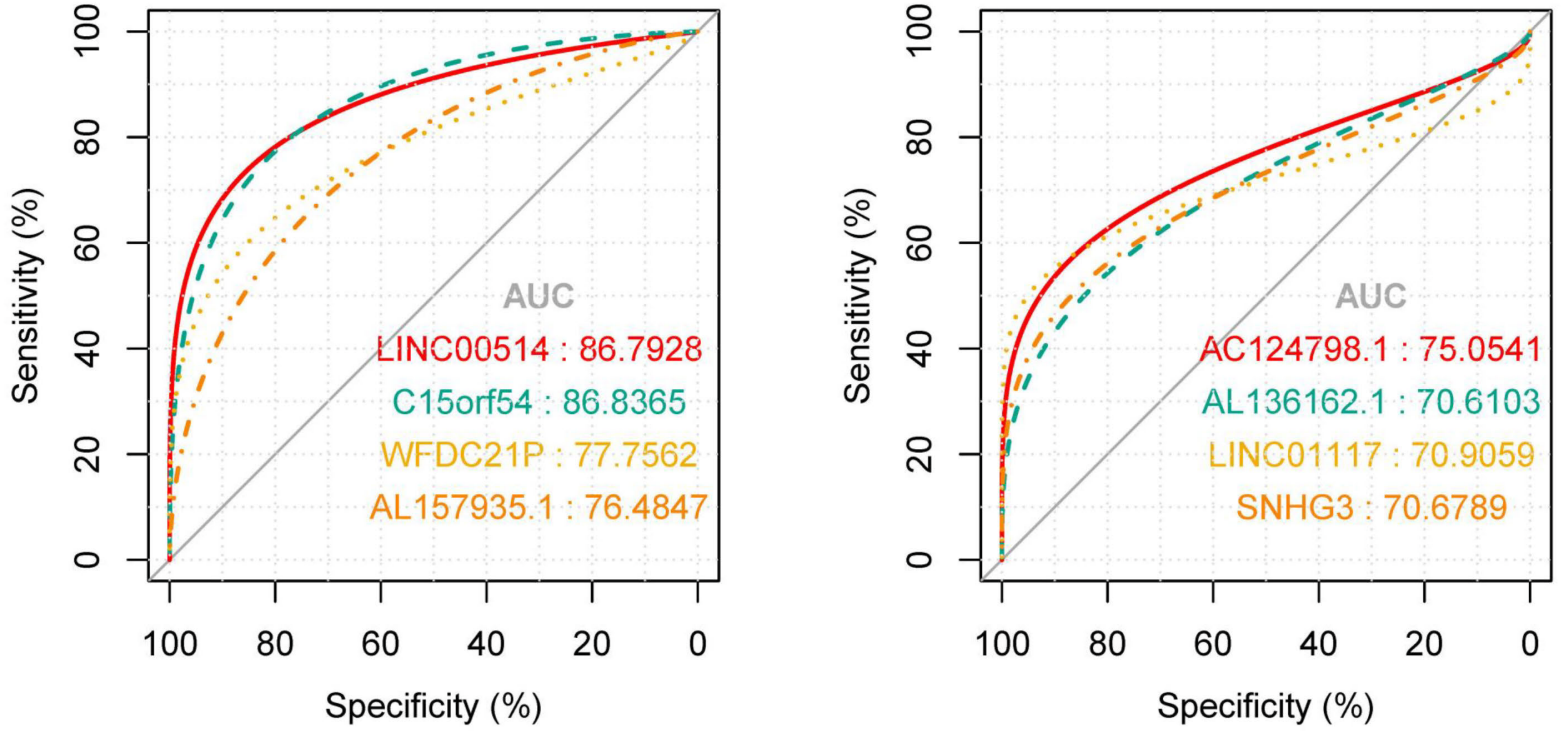
The ROC curves of key lncRNAs. The longitudinal axis shows the sensitivity of the biomarker, and the transverse axis shows the specificity of the biomarker. The AUC areas of all curves were >70%.

**Figure 7:**
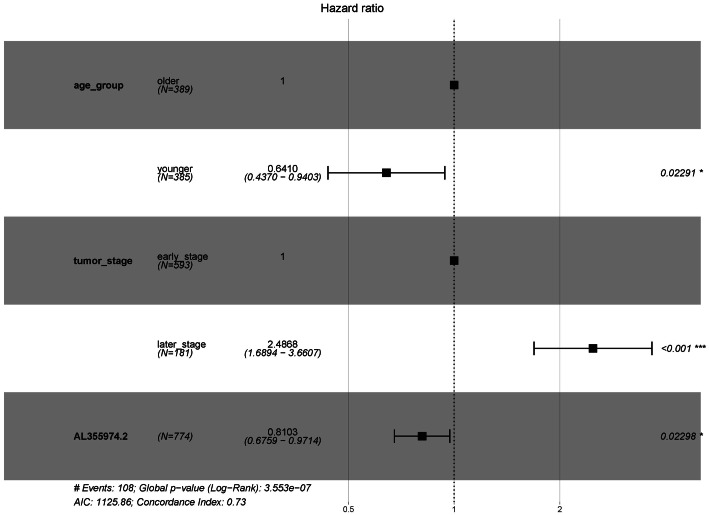
Survival analysis of key lncRNAs. The forest map shows the results of multivariate regression analysis of diagnostic age, tumor stage and AL355974.2. HR ⁄ = 1 and *p* < 0.05 were used as the criteria for screening prognostic factors.

In order to explore the diagnostic ability of the 28 candidate lncRNAs in breast cancer, the ROC curve was analysed. An AUC value >70% was set as the screening criteria; a total of eight lncRNAs were found to be able to be used for diagnosing breast cancer. Among them, the AUC values of LINC00514 (AUC = 86.7928%), C15orf54 (AUC = 86.8365%), WFDC21P (AUC = 77.7562%), AL157935.1 (AUC = 76.484%), AC124798.1 (AUC = 75.0541%), AL136162.1 (AUC = 70.6103%), LINC01117 (AUC = 70.9059%), SNHG3 (AUC = 70.6789%) were >70% (Fig. 6). In combination with the clinical information, the age of initial diagnosis, tumour stage, and the role of AL355974.2 (HR = 0.79, *p* = 0.0077) in prognosis were determined using univariate cox regression analysis (Table 4). These factors were analysed by multivariate cox regression analysis, and it was found that the early diagnosis of the tumour (young diagnosis age, early tumour stage) was of great significance for improved survival. AL355974.2 (HR = 0.8103, *p* = 0.02298) could also be used as an independent prognostic factor, and as a protective factor as its high expression helps to maintain the survival rate of patients. Our results were statistically significant (HR ≠ 1 and *p* <0.05), as shown in Fig. 7.

**Table 4:** Results of univariate regression analysis.

	coef	HR (95% CI for HR)	*p*. value
stage	0.94	2.6 (1.7–3.8)	1.90E−06
AL355974.2	−0.24	0.79 (0.66–0.94)	0.0077
age_group	−0.48	0.62 (0.42–0.9)	0.013
ZIM2AS1	0.063	1.1 (0.99–1.2)	0.11
AC124798.1	−0.11	0.9 (0.78-1)	0.14

### Extending verification of key lncRNAs

**Figure 8:**
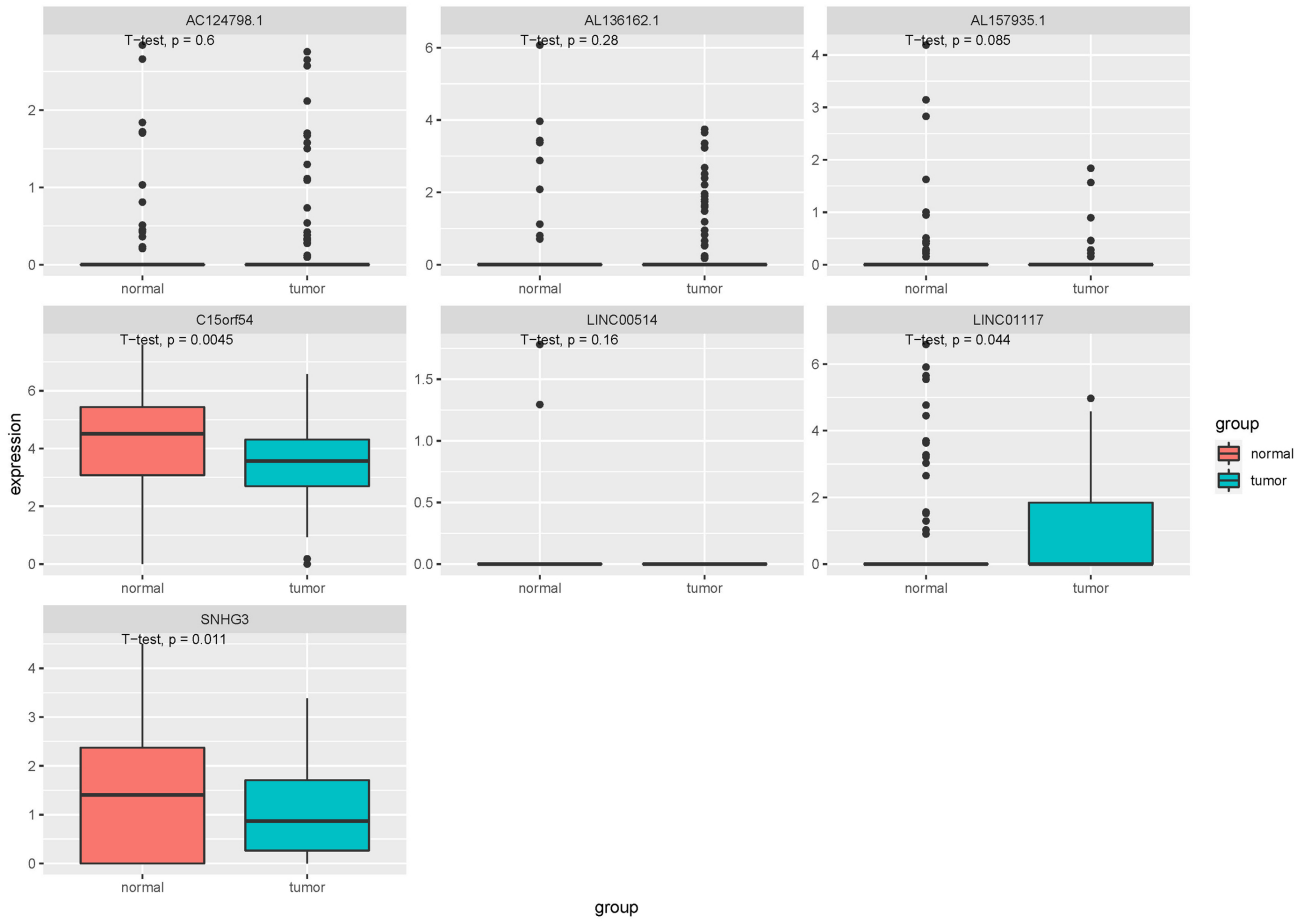
Expression of key lncRNAs in EV verification data. The vertical axis is the expression of lncRNA, which was expressed by log _2_(TPM+1), and the transverse axis is divided into lncRNA groups: the normal group and the tumor group. The *t*-test was used for comparing the expression of the two groups.

In order to further confirm the function and role of these lncRNA, we downloaded the RNA expression data in EVs and tissues of breast cancer from exoRBase (GSE93078) and GEO (GSE134359). In the EV dataset, seven lncRNAs expression data, including LINC00514, C15orf54, AL157935.1, AC124798.1, AL136162.1, LINC01117 and SNHG3 were extracted (Fig. 8). The expression levels of C15orf54, LINC01117 and SNHG3 varied between normal and tumour groups, and LINC01117 showed an up-regulated trend in tumours, while C15orf54 and SNHG3 showed a down-regulated trend in tumours. The AUC value of the seven lncRNAs were all less than 70% (Table 5), indicating that these lncRNAs do not have good diagnostic ability in the EV for breast cancer.

**Table 5:** AUC values of key lncRNA in EV verification data.

lncRNA	AUC
AC124798.1	52.1247
AL136162.1	55.2058
AL157935.1	46.8826
C15orf54	35.0998
LINC00514	49.1525
LINC01117	59.3856
SNHG3	44.0285

**Figure 9:**
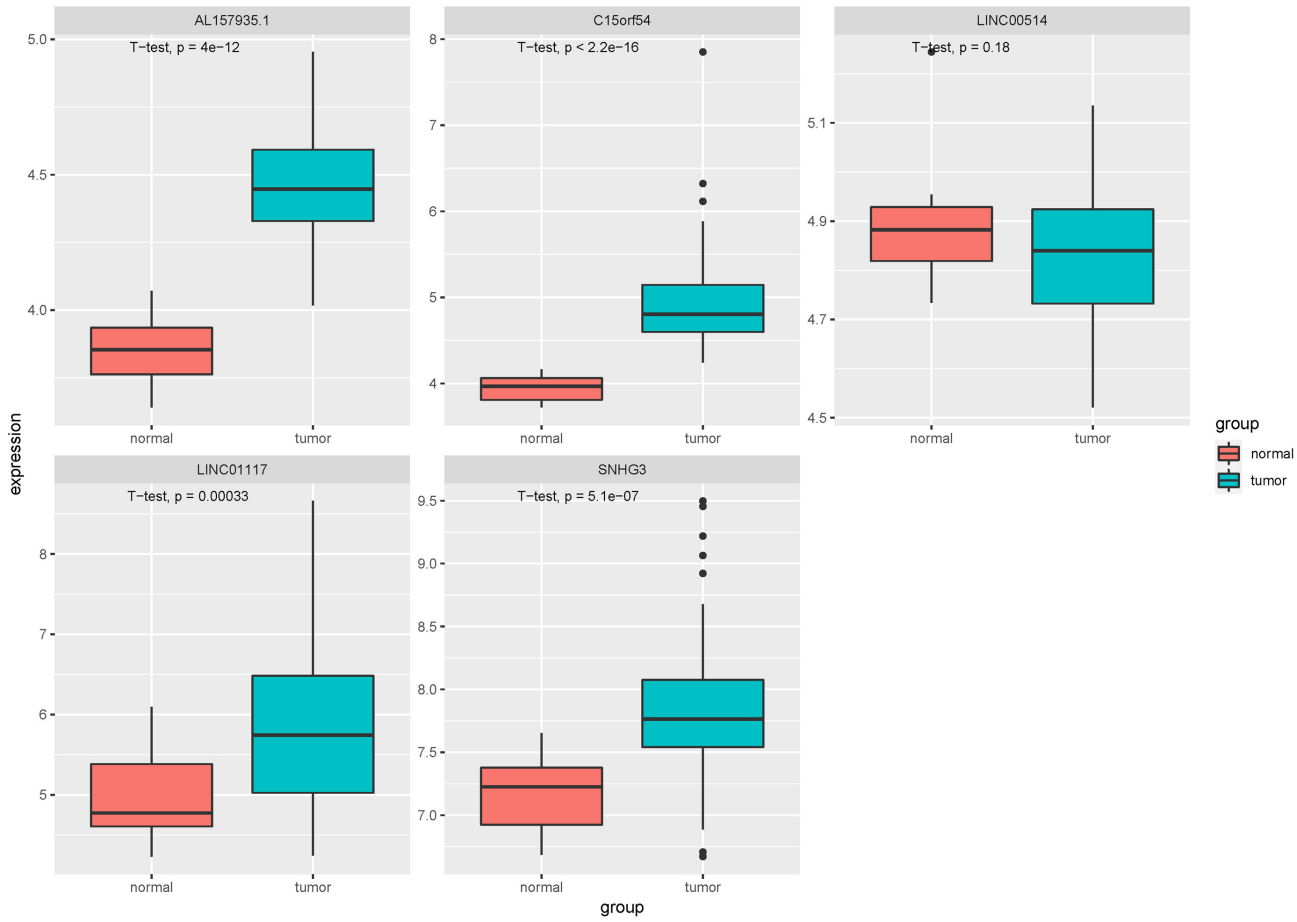
Expression of key lncRNAs in tissue verification data. The vertical axis is the expression of lncRNA, which is expressed by log _2_(TPM+1), and the transverse axis is divided into lncRNA groups: the normal group and the tumor group. The *t*-test was used to compare the expression of the two groups.

**Figure 10:**
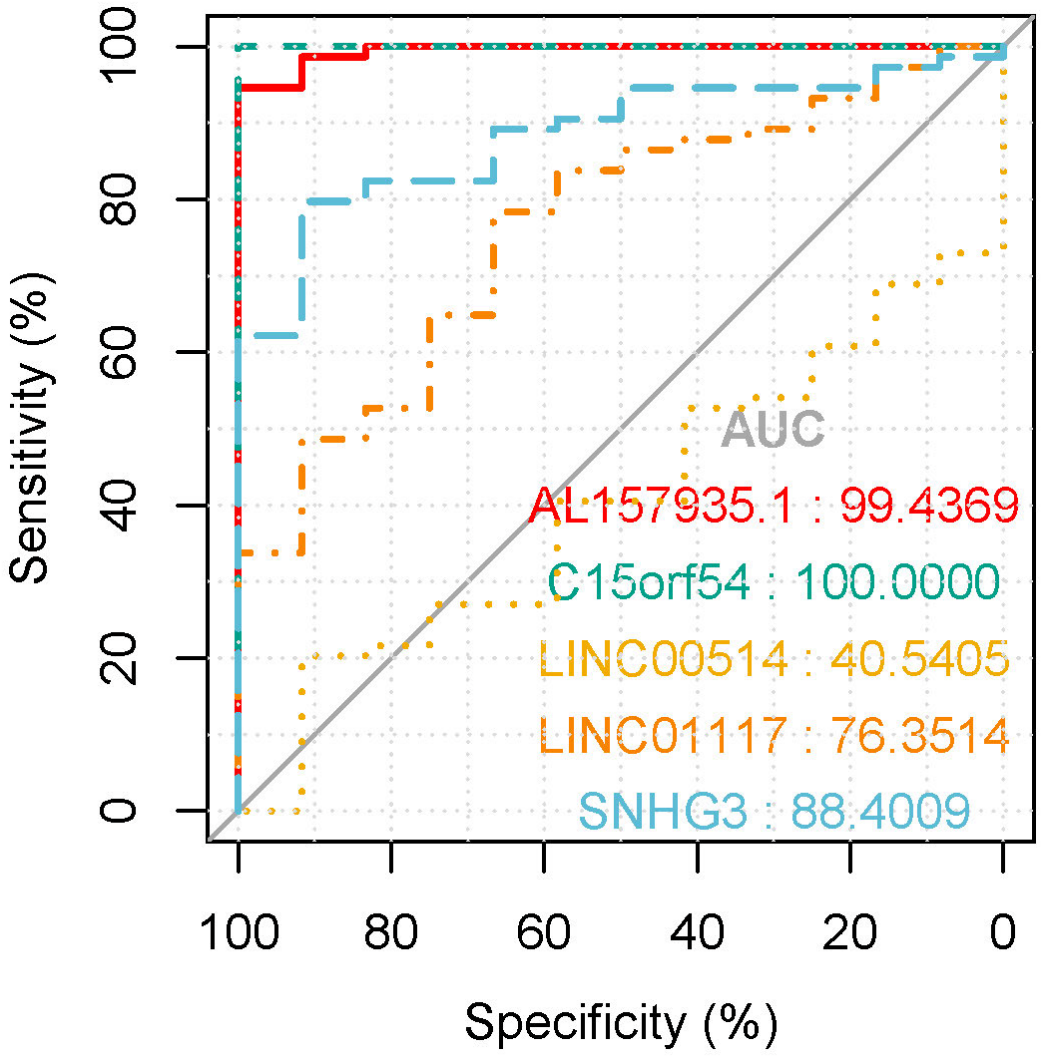
The ROC curves of key lncRNAs in tissue verification data. The longitudinal axis shows the sensitivity of the biomarker, and the transverse axis shows the specificity of the biomarker. The AUC areas of all curves were >70%.

In the tissue data set, the expression data of five lncRNAs, such as LINC00514, C15orf54, AL157935.1, LINC01117 and SNHG3 were extracted, and the expression levels are shown in Fig. 9. Among them, the expression levels of C15orf54, AL157935.1, LINC01117 and SNHG3 were different between normal and tumour groups, and all of them were up-regulated in tumours. The ROC curves of five lncRNAs were drawn, as shown in Fig. 10. LncRNAs such as C15orf54 (AUC = 100%), AL157935.1 (AUC = 99.4369%), LINC01117 (AUC = 76.3514%) and SNHG3 (AUC = 88.4009%) had good diagnostic ability in tissue samples.

## Discussion

WGCNA is a popular systematic research method in biological research. It is widely used to analyse the patterns of gene intrinsic association among different samples. Specifically, a large number of genes are identified to express similar genes and form collections, and to find gene sets and core genes that are closely related to the clinical phenotype. WGCNA has shown great value in the field of disease mechanism, classification of disease subtypes, diagnosis, and prognosis. Based on this, the expression profiles were obtained from 794 tissue samples of invasive breast ductal carcinoma and 92 cases of normal tissues from TCGA. The lncRNAs and mRNAs were separated according to annotation information. Then, plasma EVs from patients with invasive breast cancer and benign tumour was collected, and large RNAs (such as lncRNAs and mRNAs) were extracted from plasma EVs, and RNA sequencing was performed. A total of 2,450 mRNAs and 155 lncRNAs were identified by differential expression analyses. According to the trend of gene expression of plasma EVs between in breast cancer and benign tumour samples, among 155 differentially expressed lncRNAs, including 72 up-regulated and 83 down-regulated. Among them, the top 10 up-regulated lncRNAs were AC117395.3, THUMPD3-AS1, AC124798.1, LAMTOR5-AS1, AL161891.1, Z97192.2, THAP9-AS1, SNHG5, SNHG16, and MIR222HG. The top 10 down-regulated lncRNAs were MIR4458HG, AC095055.1, AC021945.1, SLC16A1-AS1, AC009414.2, ZIM2-AS1, AC007566.1, AP001011.1, LINC00608, and AC026786.2. Some studies have shown that these lncRNAs play important roles in breast cancer. For example, SNHG5 can promote the proliferation and cell cycle progression of breast cancer cells by stimulating the overexpression of proliferating cell nuclear antigen (PCNA) (Chi et al., 2019). Four lncRNAs associated with breast cancer prognosis were identified by an analysis of breast cancer gene co-expression network, including SNHG16 (Li et al., 2019). In the differentially expressed lncRNAs of breast cancer triple negative (ER, PR and Her-2 negative), human epidermal growth factor receptor 2 (HER2) positive, lumen A positive, and lumen B positive, a total of 37 lncRNAs were found to be maladjusted in four subtypes of breast cancer, and THAP9-AS1 was one of them (Li et al., 2020). This study also reflected the reliability of our data from sequencing and analysing results. The candidate lncRNAs were shown to be associated with breast cancer lesions. In order to further search for lncRNAs that could reflect the characteristics of breast cancer, lncRNAs in EVs related sequencing data from existing public databases were searched, and lncRNAs in EVs data of colon cancer, liver cancer and pancreatic cancer from the exoRBase database were downloaded. These data were then compared with the sequencing data of breast cancer from this study. A total of 37 differentially expressed lncRNAs were obtained from colon cancer samples; 69 differentially expressed lncRNAs were obtained from liver cancer samples; and 28 differentially expressed lncRNAs were obtained from pancreatic cancer samples.

A total of 41 lncRNAs were obtained from the tissue and exudate of breast cancer patients by comparing 4,879 differentially expressed lncRNAs in breast cancer tissues retrieved from the database and 149 differentially expressed lncRNAs sequenced from plasma EVs. These key lncRNAs and the corresponding targeted mRNAs were loaded into the co-expression network constructed by R-package WGCNA, and 19 gene modules were obtained. After comprehensive analysis of the modules and clinical characteristics (including age, clinical stage, survival time and status) it was found that the five modules of blue–green, yellow, purple, blue and brown were significantly correlated with clinical stage. A total of 28 lncRNAs and 3,901 corresponding target mRNAs were included. Then, the five modules were enriched by g:Profiler. Among them, the blue–green module contained 19 lncRNAs. These modules were functionally related to cancer transcriptional disorders and DNA replication. Sultan et al. (2019) found that breast cancer-related genes were mainly involved in the PPARs signaling pathway, which was associated with adipocyte differentiation, and regulating the proliferation and survival of cancer cells. Recent studies have revealed that adipocytes may enhance the proliferation and migration of breast cancer cells through PI3K-Akt-mTOR pathway (Park et al., 2020). Dong et al. (2015) found that stimulation of cAMP signal transduction could inhibit the migration of invasive triple negative MDA-MB-231 breast cancer cells. Therefore, 19 lncRNAs in the blue–green module may be involved in the regulation of proliferation and migration of breast cancer cells. The results of enrichment analysis showed that the two lncRNAs contained in the yellow module may be related to the signal exchange between breast cancer cells, cell adhesion and the ECM receptor. The blue module contains four lncRNAs, which were related to cell cycle, mitosis, and chromosome movement. This module participated in cell cycle regulation, DNA replication, homologous recombination repair and the p53 signaling pathway. These processes were very common in tumorigenesis, indicating that the functions of those four lncRNAs were related to the pathogenesis of breast cancer. The brown module contained three lncRNAs, which were involved in pyrimidine nucleotide metabolism. In addition, mRNAs expression, which was highly similar to lncRNAs expression, was extracted from this module as its potential target genes, including *TGFBR2, CAV1, AOC3, CAV2, PLIN1, EBF1* and *KIFC1*. The roles of these genes in breast cancer have been reported (Wei et al., 2015; Qian et al., 2019; Li et al., 2015), which suggested that their functions may be regulated by multiple lncRNAs. However, there was no lncRNA in the purple module, and enrichment analysis showed that they might be involved in the protein digestion and absorption process, as well as the ECM receptor interaction and relaxin pathway. In breast cancer research (Cao et al., 2013), relaxin may enhance the invasiveness of breast cancer cell lines *in vitro* by inducing the expression of matrix metalloproteinases. This link may show that the function of the purple module was related to the invasion of breast cancer cells.

In addition, the clinical information of breast cancer tissue samples was used to evaluate the clinical application of these candidate lncRNAs. The ROC curve of 28 lncRNAs in the top five modules were drawn, and eight lncRNAs with potential diagnostic markers were found to distinguish breast cancer patients from that in normal tissues. Then, the survival rates of 28 lncRNA were analyzed, combined with univariate cox regression analysis, and multivariate cox regression analysis, and identified AL355974.2, which may have a protective effect and result in a better survival rate when it is highly expressed.

In order to verify our research results, we introduced additional data sets, including the EV and tissue RNA data sets. In the EV dataset, the expression data for seven lncRNAs were extracted. Compared with the EV lncRNAs obtained by our previous experimental sequencing, there were still some differences between the two results, which may be due to the small sample size of the test sequencing. The verification data of EV revealed that there were three differentially expressed lncRNAs, however, they did not have a good diagnostic ability in EV. The tissue validation data was consistent with the exploration data we obtained from TCGA. We extracted the expression of five lncRNAs from the verification data, among which C15orf54, AL157935, 1LINC01117 and SNHG3 were differentially expressed and had good diagnostic ability in breast cancer tissues. There have been few studies conducted on these specific lncRNAs, however, there have been some studies on the function of SNHG3 in breast cancer. For example, it could promote breast cancer progression by acting as a miR326 sponge (Zhang et al., 2020). There were few samples with rich lncRNA and clinical information, but the function of AL355974.2 could not be further verified. In addition, we noticed that some lncRNAs showed an opposite expression trend in EV and the corresponding tissue samples. The cause of this is unknown and requires further study.

## Conclusions

A total of 28 breast cancer-related lncRNAs were discovered using the comparative analysis of local tissue samples and plasma EV expression profiles. Bioinformatics analysis revealed that they were related to molecular regulation of breast cancer. Among them, eight candidate lncRNAs showed a good diagnostic potential. These include LINC00514, C15orf54, WFDC21P, AL157935.1, AC124798.1, AL136162.1, LINC01117, and SNHG3. Further verification and analysis revealed that C15orf54, AL157935.1, LINC01117 and SNHG3 had better a diagnostic ability in tissue samples, but not in EV samples. In addition, AL355974.2 may an independent prognostic factor and a protective factor.

##  Supplemental Information

10.7717/peerj.13641/supp-1 Supplemental Information 1 Filter for the appropriate *β* value The suitable soft threshold was selected to show the relationship between Scale Free Topology Model Fit. Signed R ^2^ and soft threshold, also Mean Connectivity and Soft Threshold respectively.

10.7717/peerj.13641/supp-2 Supplemental Information 2 Code

## Additional Information and Declarations

### DNA Deposition

The following information was supplied regarding the deposition of DNA sequences: The transcriptome data of breast cancer tissue samples are derived from the TCGA-BRCA project at Genomic Data Commons (https://portal.gdc.cancer.gov/). dbGaP Study Accession: phs000178. The plasma exosome sequencing data of several other cancers were derived from exoRBase (http://www.exorbase.org/). The data for extending verification were from exoRBase (GSE93078) and GEO (GSE134359).

### Data Availability

The following information was supplied regarding data availability: Shu, Lirong (2022): raw_data. figshare. Dataset. https://doi.org/10.6084/m9.figshare.19329293.v1.
https://figshare.com/s/77cbcd87f63ba6010f11.
