# Supplementary figures and images for: Novel lncRNAs with diagnostic or prognostic value screened out from breast cancer via bioinformatics analyses
Source: PeerJ. 2022 Jul 14;10:e13641. doi: 10.7717/peerj.13641 (PMC9288825; doi:10.7717/peerj.13641)

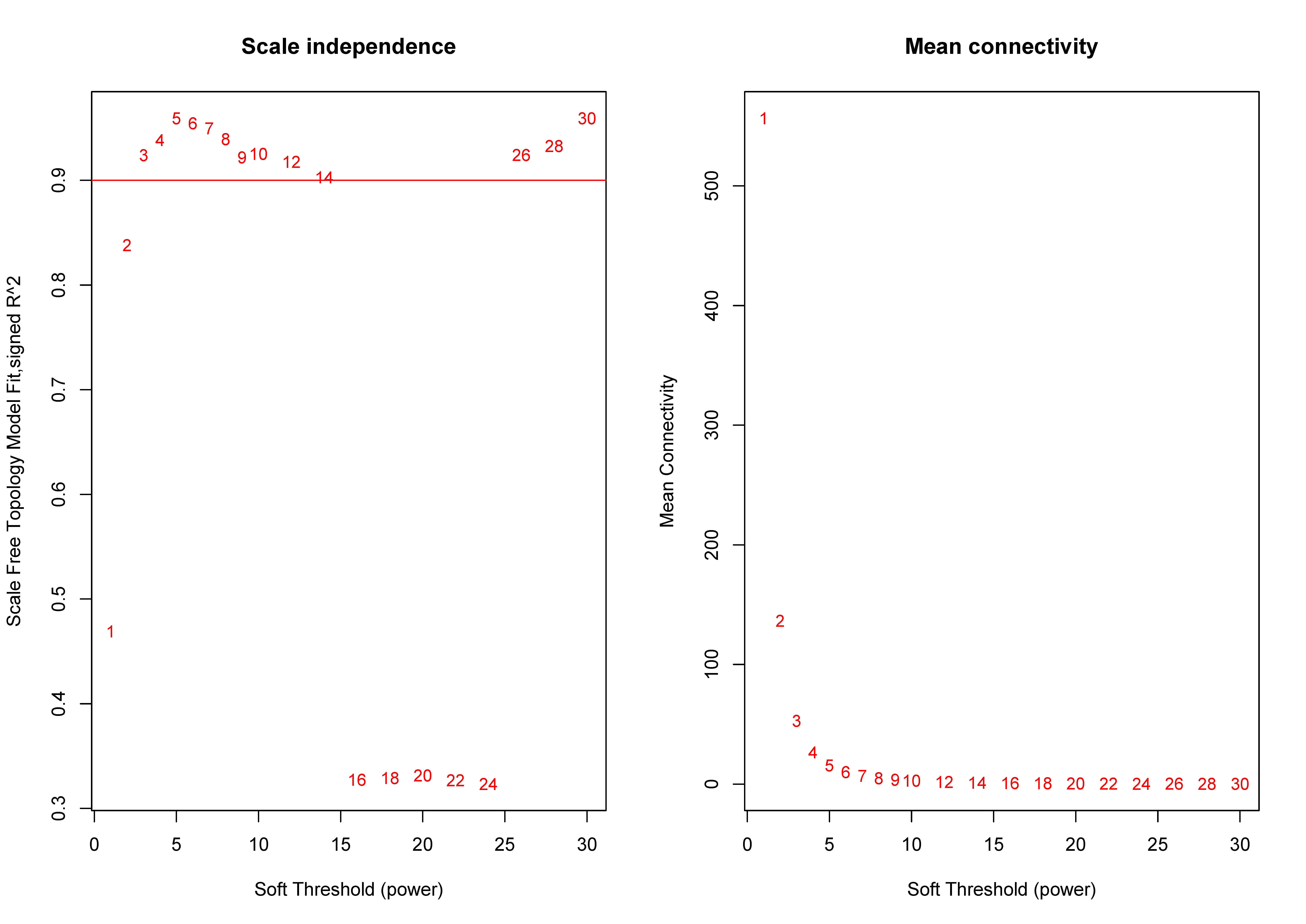

Supplement: Supplemental Information 1 — The suitable soft threshold was selected to show the relationship between Scale Free Topology Model Fit. Signed R 2 and soft threshold, also Mean Connectivity and Soft Threshold respectively. [file peerj-10-13641-s001.jpg]
